# Developing a single-session strategy for the implementation of take-home naloxone by community pharmacists using COM-B and design-thinking

**DOI:** 10.3389/frhs.2023.1227360

**Published:** 2023-08-01

**Authors:** Joanna C. Moullin, Philip Ely, Hannah Uren, Lexy Staniland, Suzanne Nielsen, Simon Lenton

**Affiliations:** ^1^ School of Population Health, Faculty of Health Sciences, Curtin University, Bentley, WA, Australia; ^2^ enAble Institute, Curtin University, Bentley, WA, Australia; ^3^ School of Design and the Built Environment, Faculty of Humanities, Curtin University, Bentley, WA, Australia; ^4^ Monash Addiction Research Centre, Monash University, Frankston, VIC, Australia; ^5^ National Drug Research Institute, Curtin University, Bentley, WA, Australia

**Keywords:** opioid overdose, naloxone, pharmacy, implementation science, theoretical models, COM-B, design-thinking

## Abstract

**Background:**

Despite the overwhelming evidence of its effectiveness, there is poor implementation of take-home naloxone by pharmacists. Barriers have been explored and mapped to the capability, opportunity, motivation—behaviour (COM-B) model of behaviour change, yet no theoretically informed implementation strategies that target known barriers have been developed. Single-session implementation strategies have been proposed as a simple, scalable way to target multiple barriers.

**Methods:**

Qualitative participatory methods, incorporating design-thinking principles, were used to develop the key messages of a single-session implementation strategy. The key messages were drafted against COM-B mapped implementation barriers identified in the literature. A participatory workshop involving a pre-mortem exercise and incorporating design-thinking principles were used to refine the messages and generate methods for dissemination. Messages were mapped to interview questions to naturally illicit stories and delivered via storytelling from a pharmacist, a general practitioner, and a person with lived experience of using naloxone.

**Results:**

A 3 minute 40 second video and a two-page printable infographic were developed and hosted on a website, with links to additional downloadable resources as a single-session implementation strategy. Email was the preferred method for receiving simple professional development communications, with social media also widely accessed.

**Discussion:**

Implementation science, behavioural change theory, and participatory design methods are a complementary combination to develop implementation strategies. Some pharmacists questioned the participatory design approach to developing an implementation strategy, as it was outside of their comfort zone. However, the participatory process involving end-users resulted in unique ideas that are unlikely to have been generated using more traditional consultative approaches. The delivery as a single-session implementation strategy allows for widespread dissemination and delivery at scale.

## Introduction

Each day in Australia, approximately 5 people die, 150 people are hospitalised, and 14 people are treated in emergency departments for opioid-related health concerns (1–3). Naloxone is a life-saving medication that reverses the effect of an opioid overdose with few side effects and no psychoactive or addictive qualities (4). Take-home naloxone (THN) is an evidence-based programme of providing naloxone and educating laypersons (e.g., people taking opioids and friends, family members, and anyone who may witness an overdose) on recognising and responding to an opioid overdose, including the administration of naloxone (4). THN saves lives and should be implemented at scale worldwide (5); however, THN programmes are poorly implemented despite the overwhelming evidence of their safety and effectiveness (6–8).

One avenue to improve the distribution of naloxone is provision of THN by community pharmacists. Due to their geographical distribution, long opening hours, role in other harm reduction services, and ability to talk with consumers without them needing to schedule an appointment, community pharmacists can reach the broad and diverse at-risk population who use illicit and/or prescription opioids. Pharmacists appear to generally support overdose education and naloxone distribution (9–12). However, a number of barriers to implementation of naloxone by pharmacists exist including the perception of the pharmacists of a lack of time, lack of awareness, workflow issues, and perceived poor comprehension by patients, stigma, training, cost, and remuneration (13–17). Implementation strategies that have been developed and tested include education programmes, academic detailing, screening tools, checklists, and pocket guides (11, 12). Strategies that address underlying stigma are needed as well as implementation strategies that may be scaled up.

In the past decade, several major barriers to implementing THN provision by community pharmacists have been removed. For example, in Australia in 2016, naloxone was down-scheduled from a prescription-only to a pharmacist-only medicine (18). This rescheduling removed a significant barrier to the implementation of THN as it enabled pharmacists to dispense naloxone without a prescription. Two other major implementation barriers were tackled in 2019 when free THN was piloted in three states of Australia (19), removing the cost barrier for patients and providing remuneration for pharmacists in the form of a dispensing fee. Unfortunately, outside of providing a dispensing fee, the roll-out of the pilot lacked additional implementation strategies for pharmacists or an awareness campaign for the public (19). Many barriers to THN access remained, including poor communication and education on THN, and harm reduction not being normalised across the sector (15). The study described here was conceived to demonstrate one approach that could be helpful in this regard following on from the work of Nielsen and Olsen (15).

Nielsen and Olsen (15) interviewed 37 Australian pharmacists to investigate the barriers to THN implementation and used the capability, opportunity, motivation—behaviour (COM-B) model to analyse their data. The COM-B model sits at the core of the behaviour change wheel (BCW), which is used to inform the development of interventions and implementation strategies by purposefully targeting identified barriers (20). The model posits that for a behaviour (B) to occur, the person needs the capability (C), opportunity (O), and motivation (M) to do so. Nielsen and Olsen (15) findings highlighted that in terms of capability, pharmacists had poor knowledge and low confidence in relation to dispensing naloxone and engaging with consumers on the topic of overdose. Regarding physical opportunity, there was poor availability of naloxone and time was a barrier, whilst social opportunity barriers related to negative attitudes towards harm reduction and a limited understanding of both naloxone's benefits and the population at risk of overdose. Pertaining to motivation, some pharmacists had negative attitudes towards people dependent on opioids and found the lack of remuneration as demotivating.

In this paper, we outline the development of a “single session for implementation strategy” to target identified implementation barriers to the provision of take-home naloxone by community pharmacists. To date, there appear to be few implementation strategies to increase the provision of THN targeting community pharmacists that are mapped to theory or identified barriers (12). Single sessions for implementation strategies are a new concept adapted from single-session patient-level interventions (21, 22). Single sessions for implementation involve a theory-led one-off encounter with a provider that can target multiple implementation determinants (22). They are “targeted, theory-informed activities aimed at promoting the uptake and sustainment of evidence-based clinical practices among clinicians” (21). In this study, we developed a short video, hosted on a website alongside an infographic and links to THN resources, as a single-session implementation strategy for THN. Furthermore, we integrate design-thinking methods into the implementation strategy development process through participatory workshops.

Whilst design-led approaches to driving healthcare innovation are widespread (23, 24), design methods are still in their infancy in implementation science, particularly with healthcare clinicians as active participants (25). In the expansive discipline of design, a number of process models and methods are human-centred (26–29). Whilst often conflated as “design thinking” (30), over 300 qualitative and quantitative methods (31) from ethnographic studies to rapid prototyping are drawn upon for problem-solving, aiming to improve project outcomes through the use of empathic, iterative, speculative, and generative methods. Such an approach to innovation does not always guarantee specific, measurable outcomes (29). By contrast, the goal of implementation is to increase the uptake of new evidence-based innovations into practice (32). Whilst implementation science and design disciplines may appear disparate, they share a common ground in their focus on finding solutions to problems or needs through human-centred, participatory methods. One such method—“co-design”—has the ability to build partners, alliances, and coalitions for action and can confirm or challenge previously held ideas and rapidly create artefacts for testing (33). We describe our process of using design-thinking with community pharmacists in combination with theoretically informed mapping of barriers to the implementation of THN provision.

In the present study, we build on the work of Nielsen and Olsen (15), with the aim to design a single-session implementation strategy targeting their identified barriers. Specifically, the study intend to engage a group of pharmacists in a design-thinking process to tackle the problem of how to encourage pharmacists to stock and dispense over the counter naloxone, and particularly, what messages would resonate with pharmacists and how to disseminate the information.

## Materials and methods

### Design

Qualitative participatory methods, incorporating design-thinking principles, were used to develop the key messages of a single-session implementation strategy. Ethics approval was obtained from Curtin University Human Research Ethics Committee (Reference number HRE2019-0816).

### Process

#### Drafting preliminary messages

First, preliminary messages were drafted by the lead researcher and programme lead at the West Australian Department of Health Mental Health Commission against the identified barriers mapped to COM components of the behaviour change wheel (15) to streamline the subsequent meeting with export stakeholders. These messages (see Table 1) were reviewed and amended at an expert stakeholder advisory group meeting (*n* = 5) consisting of subject matter experts, policymakers, and the State director of one of the leading professional societies for pharmacists.

**Table 1:** Preliminary messages from research team and adapted by expert advisory group.

Commonwealth pilot: take advantage of it	Naloxone is NOW available free in WA. Pharmacists are remunerated for supply with a dispensing fee. Five simple steps.
	Funded federal government initiative. $10 million dollars provided to pharmacy.^a^
Public health issue	All patients on S8 opioids for longer than 2 weeks are at risk.
	Half of opioid overdoses are by chronic pain patients. 70% of opioid overdoses are from prescription opioids.
	WA has the highest rate of accidental deaths related to opioid use.
Pharmacists’ role	Pharmacists have a key public health and harm reduction role, lead the country forward, be part of the change.
	Provision is part of pharmacy practice. Language is an addressable barrier.^a^
	Provision of naloxone is simple.
	Patients are relying on you—there are many reasons for overdose; patients are unaware.^a^
Myth busters	Does not increase drug use or risk-taking behaviour, but saves lives.
	No potential for abuse, available since 2004.
	Systems are changing—part of first aid courses and kits and 000 protocol. Life-saving medication like glucagon and Epipens®.
Dispensing process education	How to receive naloxone and be reimbursed (Steps 1–5).

^a^ New messages from expert advisory group.

#### Participatory meeting

Next, a 2.5-h participatory and design-thinking-oriented workshop with community pharmacists (*n *= 8) was conducted to gather ideas and perspectives on the key messages. Purposive and snowball sampling was used to recruit participants. Informed consent was obtained from all participants prior to the workshop, which was held on February 2020 at the offices of the WA branch of the Pharmaceutical Society of Australia (PSA). Workshop instructions were provided to the participants, including sharing experiences, deferring judgement, and respecting the opinions of others. The workshop was facilitated by an expert in design-thinking (PE) and a pharmacist with expertise in implementation science (JM). Data were collected through notetaking and collection of written documents and photographs (JM).

#### Activity 1: icebreaker—dissemination of information to pharmacists

After introductions and provision of background information on naloxone, the participants discussed how they receive professional news and information generally and their preferred format for education and training. This initial question acted as an icebreaker and to gauge important information on improving dissemination to pharmacists. In addition, and in line with the behaviour change wheel process for developing interventions, the pharmacists were asked to consider the key behaviours associated with the provision of THN and what were the most important for a change to occur.

#### Activity 2: pre-mortem exercise using design-thinking

A pre-mortem exercise (34) was conducted with the participants, whereby the pharmacists were posed with the problem, “Provision of naloxone is not occurring,” followed by the question, “How do we get pharmacists to stock naloxone, initiate conversations about and educate on naloxone with their patients on long-term strong opioids?” A pre-mortem exercise is a methodology that “uses prospective hindsight—a group imagines a failure and generates an explanation for it—to reduce the likelihood of the failure” (34). The pre-mortem exercise used the design-thinking iterative and cyclic phases of (i) ideation (divergence), followed by (ii) ideation (convergence), and (iii) prototyping.

*(i) Ideation (divergence):* In groups of —two to three, consisting of participants sitting at the same table, individual contributions to the pre-mortem question were written on sticky notes and placed in the middle of the table. Examples of ideas were not proposed to the participants so not to lead their thinking. The participants were asked to generate lots of ideas quickly, even ones that seemed ridiculous, to address the research question. It was explained that ideas did not need to be developed in detail and could be conferred in any form, for example, a single word, a picture, or a dot point.

To help guide and expand their thinking, the groups were given scenarios (based on COM-B) of different reasons why supply may not be occuring. Group 1 were to consider the scenario that pharmacists were uncertain about providing naloxone as it would take too long, is too complicated, may offend patients, and would condone misuse. This was based on the Motivation component of COM-B and barriers of time and seeing naloxone as a moral hazard. Group 2 were to consider that pharmacists did not know how to provide naloxone, based on the capability component of the COM-B and barriers of lack of awareness and poor knowledge. Finally, Group 3 were to consider pharmacists as being uncertain about providing naloxone as they did not feel it was their role. This scenario was focussed on the opportunity component of the COM-B and barrier of harm reduction not being normalised.

(ii) *Ideation (convergence)*: The participant groups were asked to cluster ideas into themes, select two ideas (through dot-voting or “dot-mocracy”), and to share the idea with the most votes with the other groups. Following this, *ideation (divergence) round 2:* Using a divergent idea-generating method—the “Merlin” method (35)—the participants developed new ideas from the shortlisted two on large pieces of paper, applying the principles of “enlarge,” “shrink,” “vanish,” and “reverse” on their selected idea.

(iii) *Prototyping:* One idea from each group was selected by consensus for paper prototyping.

#### Activity 3: key messages

The participants were presented with the message drafted by the expert advisory group. A group discussion was facilitated on the messages, their wording, and which they believed may shift the attitudes or intentions of the pharmacists to provide naloxone.

#### Data analysis and intervention development

First, ideas were clustered into themes, and outputs conducive to be delivered in a video or infographic were added to the key messages. A video and infographic were selected as the modes of delivery due to the COVID-19 pandemic, making face-to-face and time-consuming professional development activities inappropriate for pharmacists. Next, the intervention functions and behaviour change techniques (BCTs) were considered to try and include as many as possible in the single-session implementation strategy. Three different narrators, a pharmacist, a general practitioner, and an end-user, were chosen to provide a credible source to the messages.

An interview guide was developed whereby interview questions were mapped to the key messages, to prompt a natural delivery rather than dictation of the key messages. Campaign branding and video production were coordinated by the Make it Happen initiative of the Digital Agency Media on Mars. The dissemination plan was developed based on the responses to Activity 1, as well as what was feasible with the intervention only targeting the WA pharmacists (rather than pharmacists nationally).

## Results

Eight pharmacists attended the participatory co-design workshop: four pharmacy owners, two employee pharmacists, one country locum pharmacist, and one professional services manager for a pharmacy group. One of the attendees was an owner and board member of the WA branch of the Pharmacy Guild of Australia (membership body for pharmacy owners). Of the eight attending pharmacists, none had previously given out naloxone over the counter, and dispensing had only occurred for Doctor's Bags, as opposed to prescriptions for patients. Half of the participants had naloxone in stock in their pharmacy at the time of the workshop, and five of the eight worked in a pharmacy that is registered to provide medication-assisted treatment for opioid dependence (MATOD) (see Table 2).

**Table 2:** Sample characteristics.

Characteristic	*n* (*N* = 8)	%
Gender		
Male	3	37.5%
Female	5	62.5%
Role		
Pharmacist	2	25%
Locum pharmacist	1	12.5%
Professional services manager	1	12.5%
Pharmacy owner	4	50%
Previous OTC or prescription naloxone supply (excl. Doctors’ bag orders)		
Yes	0	0
No	8	100%
Naloxone in stock at primary pharmacy		
Yes	4	50%
No	2	50%
MATOD registration of primary pharmacy		
Yes	5	62.5%
No	3	37.5%

OTC, over the counter; MATOD, medication-assisted treatment for opioid dependence.

Initially, 10 messages were drafted against the barriers to naloxone provision by community pharmacists by the research lead and programme lead at the West Australian Department of Health Mental Health Commission and presented to the expert advisory panel. The panel adapted the messages, added three new messages and clustered them into five themes: (i) Commonwealth pilot—take advantage of it, (ii) Public health issue, (iii) Pharmacists’ role, (iv) Myth busters, and (v) Dispensing/process education (see Table 2). The final messages and information for the video, infographic, and website were mapped across all domains of the COM-B and identified barriers from Nielsen and Olsen (15) as presented in Table 3.

**Table 3:** Campaign messages mapped to the behaviour change wheel COM-B barriers identified in Nielsen and Olsen (15).

COM-B model domain^a^	Barrier^a^	Intervention function to address^a^	Behaviour change technique suggestions^a^	Included in campaign	Message
Capability	Poor knowledge and low confidence	Education, training, and enablement	Nationally consistent education*, champions, role modelling*	Yes	• Dispensing and counselling on naloxone is simple and patients are more receptive than you think • Conversation starters provided – There are some risks associated with your medicine that require first aid – Your medicine has some potential side effects which can be severe, including making you very drowsy and stopping you breathing. – Can I talk to you about how to keep safe whilst taking opioid medication and how to keep your family safe if you have opioid medication in the home? • Patients are relying on pharmacists advice – Patients may not be aware of the risk with forgetting to remove a patch before applying the next one, taking an additional dose, interactions between pain medicines and sleeping pills or other sedating medicines, mixing pain medicines with alcohol and other drugs • Counselling points: recognise, respond, prevent
	Knowledge of populations and confidence to engage	Modelling, education, environmental restructuring	Education that addresses different populations with overdose risk, *clinical prompts (e.g., in dispensing systems)*	Partial	• Naloxone is safe and easy to use • Naloxone should be available to all patients with chronic non cancer pain who are prescribed opioids and those who may witness an overdose – Pain patients on S8 opioids for over 2 weeks – CPOP patients – Fit-pack purchasing customer – Peers, friends, and family of any of the above
Opportunity—physical	Access to naloxone limited by poor availability	Environmental restructuring	*Subsidised funding model to incentivise suppliers*	Partial	• Not addressed by the campaign, however it was developed in the context on an ongoing pilot that addressed this barrier
	Physical space and time	Environmental restructuring and incentives	Consumer education materials and supports for time efficiency and confidentiality Payments to cover pharmacists’ time	Yes (together with patient materials)	• Conversation starters provided • Links to patient education materials provided on website • Pharmacists are remunerated for supply with a dispensing fee
Opportunity—social	Limited access to support on how to dispense naloxone	Environmental restructuring and enablement	Education that includes quick reference guidelines and contact information for additional support.	Yes	• Five simple steps to naloxone provision and reimbursement • Links to additional support and contact details provided on website
	Perception of limited relevant populations	Education, persuasion	Education that addresses the broad relevance of naloxone to different populations	Yes	• Pharmacists should think about naloxone every time they dispense a prescription for an opioid • Naloxone should be available to all patients with chronic non cancer pain who are prescribed opioids and those who may witness an overdose – Pain patients on S8 opioids for over 2 weeks – CPOP patients – Fit-pack purchasing customer – Peers, friends, and family of any of the above
	Harm reduction not normalised	Education, persuasion, modelling	Champions, professional leadership, social norming	Partial (through education)	• Naloxone is part of usual clinical care for people prescribed opioid • Provision is part of pharmacy practice • Patients are relying on pharmacists advice / Patients are unaware of the side effects • Reassuring for loved ones to know about naloxone and how to use it • We need to normalise conversations about naloxone • Naloxone is included in the 000 protocol for anyone to administer if they call an ambulance • Keeping naloxone in your home is like having a fire extinguisher in your kitchen, you hope you never need it but it is there just in case. • Naloxone saves lives • Naloxone is a life-saving medication (like an Epipen) • Naloxone should be kept in first aid kits and everyone know how to use it, just in case
Motivation	Negative attitudes	Education, persuasion	Education and guidelines that address different populations, normalise pharmacists role, address stigma	Yes	• All patients on S8 opioids for longer than 2 weeks are at risk • Half of opioid overdoses involve patients with chronic pain • 70% of opioid overdoses are from prescription opioids • Provision is part of pharmacy practice: duty of care for pharmacists to keep their patients safe from harm
	Disincentives—lack of remuneration	Incentivise	Funding models to support delivery at no cost to patient	Yes	• Naloxone is available for free • Pharmacists are remunerated for supply with a dispensing fee • *A Commonwealth pilot of free Naloxone is being conducted in WA* • *Register for reimbursement/Commonwealth pilot through the PPA portal*
	Disincentives—perceived “moral hazard”	Education	Increase awareness of evidence on naloxone	Yes	• Naloxone is safe and effective • Does not increase drug use or risk-taking behaviour • Naloxone has no potential for abuse • Naloxone is not new • Keeping naloxone in your home is like having a fire extinguisher in your kitchen, you hope you never need it but it is there just in case

CPOP, community program for opioid pharmacotherapy.

^a^ From Nielsen and Olsen (15).

A 3 min and 40 s video was developed. The video included interviews with a pharmacist, a general practitioner, and a person with lived experience. The messages in the video addressed barriers to implementation, while being delivered naturally, as a response to conversational questions asked by the video director. To complement the video and to address other identified barriers, a two-page printable infographic (provided in the Supplementary Material) was developed. Both the video and infographic were hosted on a Squarespace website, www.pharmacy-opioid-harm-reduction.com. The website included a list of other resources including patient leaflets, training videos, and contact details for information on the naloxone pilot and for the research team.

### Activity 1: icebreakers

Emails were the primary method that pharmacists received professional pharmacy-related information and messages. The participants reported checking their personal emails on average once per day and professional emails multiple times per day, including at lunchtime and in the evening. Emails from the Pharmacy Guild (membership body for pharmacy owners) were most read by pharmacy owners. Daily emails by the Australian Journal of Pharmacy (AJP) were popular with other daily “pharmacy news” emails skimmed or looked at occasionally (e.g., Pharmacy News and Pharmacy Daily). Emails from the Professional Society for Pharmacists (PSA) were read for training-related information and guidelines, and emails from indemnity insurance, wholesalers, and pharmacy banner/staff emails were also mentioned. In addition, pharmacists received information from flyers, fax, websites, social media, podcasts, service providers, pharmaceutical representatives, and word of mouth. Social media was used professionally by six of the eight participants, with Facebook being most popular, followed by Twitter, LinkedIn, and Instagram. Podcasts were listened to for professional information by three attendees.

Pharmacists identified four behaviours as being roadblocks to the implementation of THN: stocking naloxone, initiating conversations with patients, educating patients, and signing up for the Commonwealth pilot. Identifying patients, dispensing naloxone, providing educational materials, patient follow-up, and educating other staff were not prioritised by the participants, although knowing where to go for educational materials was flagged as important.

Obtaining naloxone stock has been an issue and is crucial for the THN provision to become routine (15). The participants felt that the topic was sensitive and expressed a need for training on initiating conversations and appropriate language to not accuse patients. They liked the idea of calling THN a “first aid measure” as it would “make life a lot easier to start conversations.” Using the term “side effect” was also perceived as easier and more acceptable than “overdose.” Similarly, educating patients was raised as a barrier due to lack of knowledge. There was a desire for a cheat sheet or flyer targeting consumers for pharmacists to give out, or a poster in the pharmacy to normalise talking about naloxone. Signing up for the pilot was considered a major issue, due to a lack of awareness of the programme.

### Activity 2: ideas emergent from design-thinking pre-mortem

During the design-thinking workshop, the participants were able to think beyond the scope of the initial problem statement. Themes from generated ideas were related to stock, patient groups, patient education/awareness, pharmacist education, messaging/language, and system changes. The generated ideas are presented in Table 4.

**Table 4:** Ideas for increasing the implementation of THN in community pharmacy.

Theme	Ideas
Stock	Wholesalers to send flyer when in stock; Credit provided for expired stock; Free to pharmacy instead of pay then claim back (i.e., no loss); Give kit for free in stock boxes with training disc.
Patient groups	All new CPOP patients provided with naloxone; Tell patients as part of a medication review or MedsCheck; Tell aged care facilities, local drug dealers, shopping centre security, local police, local doctors.
Patient education/awareness	Consumer education so patients initiate, e.g., include in first aid courses, information provided by drug and alcohol services, flyers, posters, ask about naloxone messaging in/on product boxes, naloxone information on opioid consumer medicine information leaflets, flyers for fit-packs (needle and syringe packages supplied free); Campaign, potentially initiated by Government, to normalise and educate on naloxone (including social media, TV, bus stops); conversation tools to use with clinicians.
Pharmacist education	Continuing professional development (CPD) programme; Updates on trial to encourage and share what is working; Add to CPOP training; Add to Intern Training Programme (ITP); Pharmaceutical representative for in-store training; Sessions at local conferences; Training from professional societies; Flyer from wholesaler to owner; messages to target accountability, fear, lived experience; Podcast and video on (i) Why this medication is needed. (ii) How to have the conversation, e.g., role-play/demonstrations.
Messaging and language	Side effects rather than overdose; “first aid” for your medicine; messages to target accountability, fear, lived experience; Making it part of the process, no thought; Casual conversation starters (e.g., “You’re getting your pain medicine, do you have naloxone?”).
Support	Guideline/checklist to make the process easy; Easy to read email of how to order, were to go for training and how to claim reimbursement.
System changes	Dispensing software pop-up; Promotion of dispensing fee; Prompt on dispense system for no opioid dispensing for a patient; Larger dispensing fee.

CPOP, community program for opioid pharmacotherapy; CPD, continuing professional development.

Three ideas were taken forward for the second round of ideation and prototyping. Idea #1 considered including naloxone as part of Medication Review consultations. This provides additional time and reimbursement for having the conversation about naloxone every 12 months. Idea #2 was to normalise the conversation by attaching naloxone to the opioid medication packaging. Concepts within this idea ranged from Velcro-sticking the two items together, to a message inside the box to be taken to the pharmacist to receive naloxone, or a sticker to be placed on the prescriptions of the doctors to encourage collaboration (co-prescribing). Idea #3 was the provision of free stock to pharmacies or include free stock in first aid kits and providing free “first aid kits” for patients who live with pain (medication and naloxone in a bag).

### Activity 3: key messages

The participants highlighted the key messages. Firstly, “naloxone is now available for free and pharmacists are remunerated for supply with a dispensing fee. Only takes five simple steps,” as they thought there was a general lack of awareness of the Commonwealth pilot and the process for supplying naloxone. Second, “all patients on S8 opioids for longer than two weeks are at risk,” corresponding to poor knowledge around relevant populations. Third, “provision of naloxone is simple, language is an addressable barrier,” together with “pharmacist have a key public health and harm reduction role” were raised due to the need to normalise naloxone for people taking opioids to relieve pain, and finally, the myth busters to normalise naloxone and reduce stigma “naloxone is part of first aid courses, kits and 000 protocols. It's a life-saving medication like glucagon and Epipens®” and “Naloxone does not increase drug use or risk-taking behaviour but saves lives.”

## Discussion

This project was built on the work of Nielsen and Olsen (15) to develop a theoretically informed, participatory designed, single-session implementation strategy that aimed to increase the implementation of THN by community pharmacists.

The implementation strategy consisted of a video that includes the intervention functions of education, modelling, persuasion, and promoting available incentivisation and linked BCTs. The video addressed known barriers, provided education for pharmacists on opioid-related overdose, naloxone, identifying at-risk patients, delivering advice, stigma and discrimination, and included content delivered by champions and role models from the pharmacy and the affected community (individuals who use opioids, peers, and those likely to witness an overdose). Some intervention functions and techniques were not appropriate or feasible due to the format of the implementation strategy being a communication campaign. These intervention functions included environmental restructuring, training, and enablement and BCTs such as clinical prompts, as well as those associated with the system level barrier of stock availability. This is consistent with the proposition that single-session implementation strategies may address multiple implementation barriers in a scalable and efficient manner (21); however, additional multilevel strategies are likely to be also required to target all determinants of change across all levels.

The design-thinking process resulted in creative and valuable ideas, but was uncomfortable for some pharmacists. These pharmacists spoke directly with researchers and were noted in the field notes about seeing the activities as futile and not initially seeing the merit in their “crazy” ideas. However, the idea from the activity of “expanding their idea” (using the Merlin method) led one group suggesting for naloxone to be provided for free in all first aid kits. With a little tweaking, this became a key message. As many healthcare professionals are not accustomed to open-ended creative inquiry, we suggest that additional time be provided to acknowledge and normalise all reactions to the process up-front, allowing these to be noted before moving on, in addition to providing examples of the potential impact of such processes (36). We also encourage researchers to engage in these methods to extract ideas that may not come from a simple or prescribed/pre-determined line of questioning: the reasoning logic of design-led approaches is largely abductive (37), focussing on the generative possibilities of “what if?” (36).

The participatory nature of the workshop also allowed the participants to share some general ideas and demographic information including background on naloxone supply. For example, the participants shared that an educational flyer is provided for “Fit-packs” (needle and syringe packs in hard disposal containers sold in pharmacies for people who inject drugs), but not for other opioids, whilst others raised their concerns about doctors not engaging in the space and prescribing naloxone. Furthermore, supply issues were raised along with suggestions to increase Health Department-issued communication to increase awareness, targeting owners and employee pharmacists, and discuss naloxone with all patients who use opioids, including prescribed and elicit forms. These ideas have the potential to be included in subsequent implementation strategies.

## Limitations

This study was conducted at the onset of the COVID-19 pandemic and a feasible, acceptable, and appropriate mode of delivery for an implementation strategy was required given this context and the additional pressures and workload being placed on community pharmacists. As a result, the researchers and expert advisory group pre-determined, prior to the participatory workshops and placed as a boundary on the intervention, to develop a single-session implementation strategy through a video format. In addition, being a single-session implementation strategy, it was decided that the intervention was to target pharmacist and pharmacy level barriers, as these contained the most barriers in the literature, rather than be a complex multilevel implementation strategy. These boundaries placed a limitation on addressing all barriers identified in the preceding work, in particular those at a system level (e.g., stock availability) as well as consumer/end-user level influences. Another limitation of this study was the lack of involvement of consumers/end-users in the development workshop. This was due to a difficulty in recruiting end-users but was mitigated to some degree in production by the final messages being real responses from an affected end-user.

Finally, a second workshop was planned for pharmacists, but with the onset of the COVID-19 pandemic and the workload increase for pharmacists, it was not feasible to conduct this workshop. Whilst the first workshop (as described in this paper) ideated broadly about the strategies to address naloxone not being provided (regardless of the mode of delivery), the second workshop was to focus the activities on the messages that may address the problem in a video intervention. The implication of this was that the research team needed to determine the ideas that were reasonable to be delivered in the intervention.

## Future work

As of 1 July 2022, due to the success of the pilot in three Australian States, the Australian Government announced that $19.6 million would be invested over the subsequent 4 years to implement free take-home naloxone in the country (38). This produces both an opportunity and challenge. Whilst the single-session nature of the intervention video is feasible for a national scale-up, there is a need for future studies to evaluate its implementation and impact.

Implementation science, behavioural change theory, and participatory design methods are a complementary combination to develop implementation strategies. Together, they can address theoretically and empirically derived implementation barriers and create unique ideas that are unlikely to have been generated using more traditional consultative approaches. Furthermore, it appears that multiple implementation barriers can be addressed in a single-session implementation strategy.

## Data Availability

The raw data supporting the conclusions of this article will be made available by the authors, without undue reservation.
