# Supplementary material for: Developing a single-session strategy for the implementation of take-home naloxone by community pharmacists using COM-B and design-thinking
Source: Front Health Serv. 2023 Aug 1;3:1227360. doi: 10.3389/frhs.2023.1227360 (PMC10434522; doi:10.3389/frhs.2023.1227360)
Supplement: Supplementary file 1 [file Image1.pdf]

Harm Reduction with Prescription Opioids

Naloxone can prevent serious side effects from opioid use.

Naloxone is available free and pharmacists are remunerated for OTC supply with a dispensing fee.

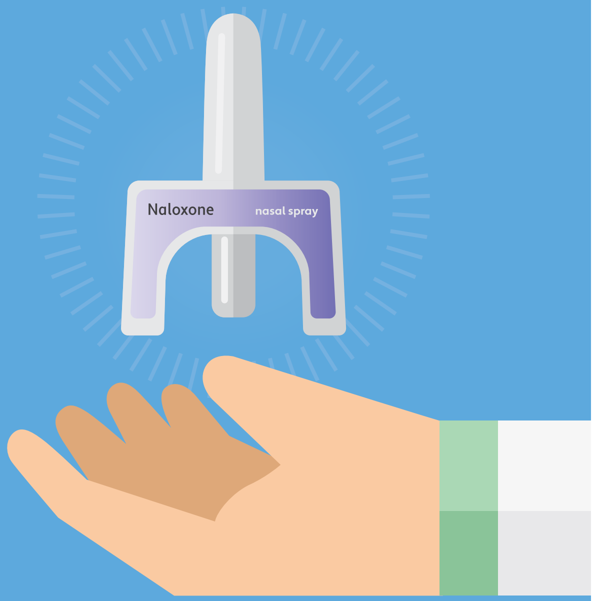

Naloxone should be available to all patients with chronic non-cancer pain who are prescribed opioids and those who may witness an overdose.

- Pain patients on S8 opioids for over two weeks
- CPOP patients
- Fit-pack purchasing customer
- Peers, friends and family of any of the above

Did you know...?

Naloxone is a first aid measure, just like an Epipen, but now available also as a nasal spray.

50% of prescription opioid overdoses involve people with chronic pain

Naloxone is safe, effective and does not increase drug use or risk taking behaviour

Naloxone is included in the 000 protocol for anyone to administer if they call an ambulance

Naloxone is part of usual clinical care for people prescribed opioids

Five simple steps to order naloxone and be reimbursed

1. Register for the Take-Home Naloxone Pilot through PPA website
2. Order naloxone through your wholesaler
3. Offer naloxone to patient or person who may witness an overdose
4. Supply and educate on naloxone (up to two packs per consultation)
5. Enter data and claim payment through PPA portal

Naloxone saves lives.

Curtin University Human Research Ethics Committee (HREC) has approved this study (HREC number HRE2019-0816). Should you wish to discuss the study with someone not directly involved, in particular, any matters concerning the conduct of the study or your rights as a participant, or you wish to make a confidential complaint, you may contact the Ethics Officer on (08) 9266 9223 or the Manager, Research Integrity on (08) 9266 7093 or email hrec@curtin.edu.au.

Initiate the conversation

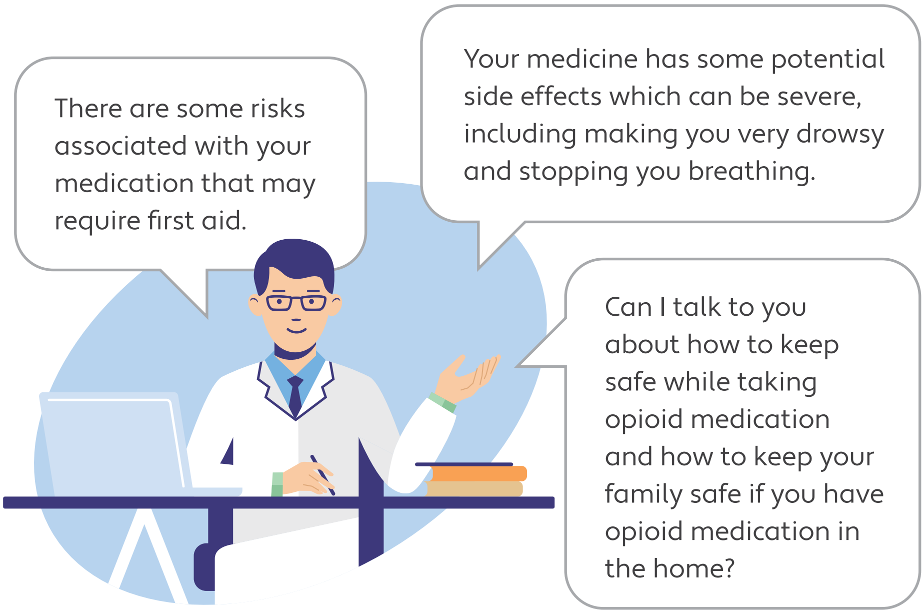

Patients may not be aware of the risk with:

- Forgetting to remove a patch before applying the next one
- Taking an additional dose
- Interactions between pain medicines (including patches) and sleeping pills or other sedating medicines
- Mixing pain medicines with alcohol and other drugs

Patients are relying on your advice

Counselling points

1. Recognise (signs for when naloxone is required)

A) Unresponsive, or heavily sedated, body is limp/slumped
B) Snoring or gurgling noises (sounds just like sleep apnoea)
C) Irregular, shallow or no breathing
D) Appearance changes: small pupils, grey/blue lips, cold clammy skin

2. Respond

A) Call 000 (do not hang up, 000 will talk you through how to give naloxone)
B) Administer naloxone (see packet insert for simple steps)
C) Stay with patient
D) Administer a second dose, if no response in two to three minutes (you can give more naloxone at any time) and if still no response start CPR

3. Prevention

A) Avoid combining your pain medication with other medications, including alcohol and opioid patches, without checking with your Doctor or Pharmacist
B) Make a note of when you took your medication as it is easy to forget
C) Contact your Doctor or Pharmacist if you are still in pain before taking more medication

Keeping naloxone in your home is like having a fire extinguisher in your kitchen, you hope you never need it but it is there just in case.

Naloxone saves lives.

Curtin University Human Research Ethics Committee (HREC) has approved this study (HREC number HRE2019-0816). Should you wish to discuss the study with someone not directly involved, in particular, any matters concerning the conduct of the study or your rights as a participant, or you wish to make a confidential complaint, you may contact the Ethics Officer on (08) 9266 9223 or the Manager, Research Integrity on (08) 9266 7093 or email hrec@curtin.edu.au.
